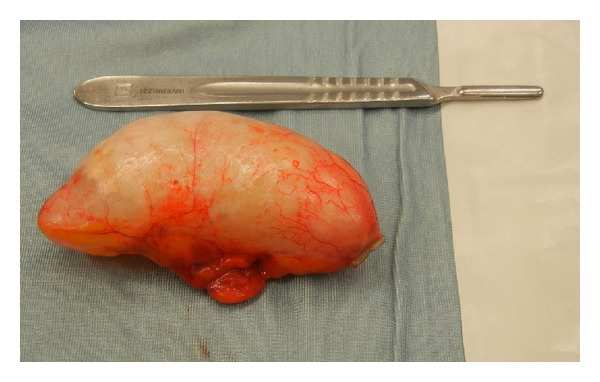# Large Mucocele of the Appendix at Laparoscopy Presenting as an Adnexal Mass in a Postmenopausal Woman: A Case Report

**DOI:** 10.1155/2014/486078

**Published:** 2014-04-06

**Authors:** Elvira Paladino, Maria Bellantone, Francesca Conway, Francesco Sesti, Emilio Piccione, Adalgisa Pietropolli

**Affiliations:** School of Medicine, Academic Department of Biomedicine & Prevention and Clinical Department of Surgery, Section of Gynecology and Obstetrics, Tor Vergata University Hospital, Viale Oxford 81, 00133 Rome, Italy

## Abstract

A 79-year-old female was referred to our Gynecologic Department presenting with a pelvic magnetic resonance imaging (MRI), showing an adnexal mass, later confirmed at the pelvic examination. The patient's routine laboratory tests were normal. A sonographic examination was performed with inconclusive results. Although the ultrasonography excluded the presence of vascularization and malignant degeneration, the adnexal localization appeared to be dubious. The laparoscopy and the subsequent histologic examination revealed the presence of a mucocele of the appendix. The following case report focuses the attention on a misdiagnosis of appendiceal mucocele. The misdiagnosis caused no negative impact on the treatment that in this case was adequate and successful.

## 1. Introduction

Mucocele of the appendix is a rare pathology with an incidence of approximately 0.2 to 0.3% of all appendicectomy specimens [1–4]. Mucocele of the appendix was first described by Rokitansky in 1842. Today, the modern classification defines four subgroups of mucoceles: a simple retention cyst determined by intraluminal accumulation of mucoid material, rarely greater than 2 cm; mucosal hyperplasia, a mild dilatation with areas of hyperplastic epithelium; mucinous cystadenoma characterized by a dilatation of the lumen up to 6 cm with low grade dysplasia; mucinous cystadenocarcinoma with stromal invasion and intraperitoneal spread, similar to that of ovarian mucinous cystadenocarcinoma. The symptomatology of mucoceles is not specific and sometimes they can be asymptomatic [3–8].

## 2. Case Presentation

**Figure 1:**
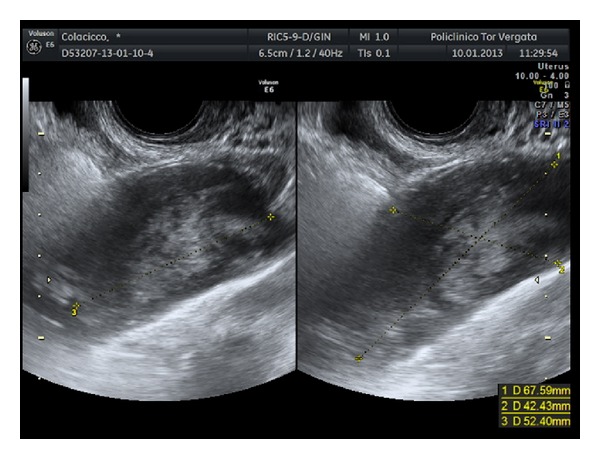


A 79 year-old woman was referred to our department with a magnetic resonance imaging (MRI) requested during her last gynecological examination, when a pelvic mass had been incidentally detected by office ultrasonography. The MRI showed a well capsulated cystic mass on the right ovary with a maximum diameter of 8 cm, homogeneous fluid content, and smooth regular walls without inner vegetations. The patient did not complain of any symptoms and her clinical history was characterized only by episodes of atrial fibrillation. No documentation of previous surgeries was reported. The pelvic examination was negative except for the presence of a palpable mass appreciated at the right vaginal fornix. Laboratory tests were all negative as well as tumor markers (Cea, Ca125, Ca15.3, and Ca19.9 were 1.26 ng/mL, 8.10 U/mL, 14.10 U/mL, and 3.44 U/mL, resp.). The sonographic examination pointed out the presence, in the right adnexal region, of an oblong, well capsulated, uniloculated mass, characterized by dishomogeneous content, distal shadowing and not vascularized at power Doppler evaluation (Figure 1). The mass appeared fixed on the surrounding planes at dynamic assessment. The left ovary seemed normal, whereas it was not possible to localize the parenchyma of the right ovary. A laparoscopic exploration was then performed: the uterus, the ovaries, and the fallopian tubes were all negative, and an appendiceal mass with a maximum diameter of 9 cm was found (Figure 2), loosely adherent to the surrounding planes with no signs of periappendiceal inflammation or free fluid. Considering the findings of the laparoscopic exploration, the negativity of tumor markers, and the ultrasonographic picture suggestive of a benign pathology, the treatment was a laparoscopic appendicectomy. The surgeon performed a section of the mesoappendix, removed the entire specimen using an endobag, and expanded the breach of the right trocar. The histopathologic diagnosis was appendiceal mucocele. The gross description of the appendix (9 × 4 × 3.5 cm in size) showed diffuse dilatation, a copious amount of mucus, and focal wall thickenings with calcifications. The microscopic description showed acute and chronic inflammatory cell infiltration within the wall of the appendix, presence of gigantocellular cells, and focal areas with mucus covered by simple epithelium. The patient was discharged on the first postoperative day.

## 3. Discussion

The clinical presentation of an appendiceal mucocele is not specific, with inconstant presence of abdominal and pelvic pain, nausea, and fever. Often, appendiceal mucoceles are asymptomatic [7–9]. It may be interesting to consider that the diagnosis of appendiceal mucocele is likely to take place during a gynecological ultrasound, and that its sonographic appearance can vary widely from a cystic lesion with anechoic content to a complex hyperechoic mass. When focusing on appendiceal mucoceles mimicking ovarian cysts, in spite of the ultrasonographic variability, the majority would probably be classified as benign [10]. The primary key to sonographically differentiate the mucocele from a case of uncomplicated appendicitis is the lack of appendiceal wall thickening of more than 6 mm [11, 12]. This distinguishing feature produces a typical target lesion, characterized by an echogenic submucosal layer sandwiched by the inner hypoechoic lamina propria/muscularis mucosa and outer hypoechoic muscular layer [1]. Appendiceal mucocele appears as a solid mass with a bottle-like or banana-like appearance, sliding freely over the uterus and ovaries [13, 14]. However, a highly specific ultrasonographic marker of an appendiceal mucocele is the “onion skin sign” (concentric echogenic layers with septa and fine echoes) [15]. In fact, according to Caspi et al., the presence of this sign within a cystic mass in the right lower abdominal quadrant, with a normal right ovary, may be specific for the diagnosis of appendiceal mucocele. Computed tomography (CT), MRI, colonoscopy, and barium studies are all useful additional examinations. For example, colonoscopic findings, including the “volcano sign” (the appendiceal orifice seen in the center of a firm mound covered by normal mucosa) and a bulbous submucosal lesion of the cecum, enable accurate diagnosis and can direct management [16]. On MRI, the lesions are well encapsulated cystic masses, hyperintense on T2-weighted sequences, and hypo- or isointense on T1-weighted sequences. The typical CT appearance of an appendiceal mucocele is a large and well-encapsulated cystic mass in the expected region of the appendix; calcifications of the cyst wall are highly specific to this lesion and a useful feature for differentiating the cyst from an abscess [1, 17, 18]. Usually, CT is considered the most informative imaging technique although the diagnosis is more difficult when calcifications are absent and it can fail to identify the organ of origin, as demonstrated in the literature [3, 19, 20]. In the case of giant mucoceles, the diagnostic problem may be even more challenging because of the difficulties in defining the precise anatomic relationship with the cecal region and also for the fact that most CT findings are insensitive [21].

Finally, the preoperative diagnosis is the major component to prevent and to minimize intraoperative and postoperative complications, such as intussusception, bleeding, peritonitis, and pseudomyxoma peritonei [3, 4, 10, 15]. Moreover, appendiceal mucinous tumors have been reported to coexist with ovarian epithelial tumors, especially of mucinous type, in association with pseudomyxoma peritonei, even if the pathogenetic relationship between these tumors remains obscure. In fact, during ovarian cancer surgery, appendicectomy is frequently required to rule out the presence of microscopic metastasis or of a primary appendiceal cancer and to achieve optimal cytoreduction. Coexistence with colon adenocarcinoma and endometrial adenocarcinoma has also been reported in the literature, although rarely [20, 22, 23]. A careful preoperative and intraoperative evaluation are required considering the mucocele's potential to develop cancer, the risk of rupture that may lead to the catastrophic complication of pseudomyxoma peritonei, and the documented association with coexistent pathology [10, 15, 16].

Regarding the treatment, in general, an appendicectomy is advised for mucosal hyperplasia and cystadenoma with intact appendiceal base; cecum resection is indicated for cystadenoma and right hemicolectomy in case of cystadenocarcinoma with or without lymphadenectomy [16, 24]. An algorithm for selecting the type of surgery has been provided by Dhage-Ivatury and Sugarbaker, based on the possible risk of perforation, involvement of margins of resection, and lymph nodes in the mesoappendix. Indications and contraindications to laparoscopic surgery continue to be redefined, since all appendiceal tumors can result in diffuse peritoneal implantation and dissemination of cancer that may be associated with laparoscopic resection of structures containing a malignancy. Therefore, some authors recommend an open surgery that has the advantage of the release and the exteriorization of the cecum, avoiding contamination of the cavity in case of accidental rupture of the appendix [24, 25]. Conversion to laparotomy should be considered if the lesion is traumatically grasped or if the tumor clearly extends beyond the appendix or if there is evidence of peritoneal malignancy [16, 24, 26].

In conclusion, currently, the best surgical technique remains controversial and laparoscopic appendicectomy is not contraindicated in mucocele of appendix, if appropriate precautions can be taken intraoperatively to avoid rupture in the peritoneal cavity [18, 26, 27].

In our patient the mucocele was incidentally reported to mimic an adnexal cyst by the MRI, while the ultrasonography failed to determine the origin of the mass. Our diagnostic error was due to three factors: the onion skin sign was not evident, probably because it was not properly investigated due to the fixity of mucocele; calcifications within the mass and the wall were the predominant sonographic marker; the right ovary was not recognized. The surgeon recognized this pathological entity at the time of surgery and performed a simple appendicectomy, without perforation and with no discharge into the peritoneum. No pathologic process at the basis of the appendix was found and the margins of resection were negative.

Therefore, according to the literature, no long-term followup of the patient was necessary.

## Figures and Tables

**Figure 2:**